# *N*′-(3-Phenyl­allyl­idene)nicotinohydrazide monohydrate

**DOI:** 10.1107/S1600536809043001

**Published:** 2009-10-23

**Authors:** R. Archana, N. Saradhadevi, A. Manimekalai, A. Thiruvalluvar, R. J. Butcher

**Affiliations:** a PG Research Department of Physics, Rajah Serfoji Government College (Autonomous), Thanjavur 613 005, Tamil Nadu, India; b Department of Chemistry, Annamalai University, Annamalai Nagar 608 002, Tamilnadu, India; c Department of Chemistry, Howard University, 525 College Street NW, Washington, DC 20059, USA.

## Abstract

In the title compound, C_15_H_13_N_3_O·H_2_O, the dihedral angle between the pyridine and phenyl rings is 35.45 (7)°. Inter­molecular O—H⋯O, O—H⋯N, N—H⋯O and C—H⋯O hydrogen bonds are found in the crystal structure. In addition, C—H⋯π inter­actions involving the pyridine and phenyl rings are also found.

## Related literature

For a related crystal structure and its chemical and biological applications, see: Archana *et al.* (2009 ▶).
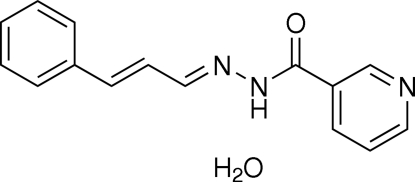


## Experimental

### 

#### Crystal data

C_15_H_13_N_3_O·H_2_O
*M* *_r_* = 269.30
Monoclinic, 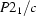

*a* = 9.8456 (3) Å
*b* = 9.1288 (3) Å
*c* = 15.5389 (5) Å
β = 95.938 (3)°
*V* = 1389.12 (8) Å^3^
*Z* = 4
Cu *K*α radiation
μ = 0.72 mm^−1^
*T* = 110 K
0.48 × 0.45 × 0.24 mm

#### Data collection

Oxford Diffraction Xcalibur, Ruby, Gemini diffractometer
Absorption correction: multi-scan (CrysAlisPro; Oxford Diffraction, 2009 ▶) *T*_min_ = 0.704, *T*_max_ = 1.000
6007 measured reflections
2742 independent reflections
2346 reflections with *I* > 2σ(*I*)
*R*_int_ = 0.022

#### Refinement

*R*[*F* ^2^ > 2σ(*F* ^2^)] = 0.043
*wR*(*F* ^2^) = 0.125
*S* = 1.05
2742 reflections
193 parameters
H atoms treated by a mixture of independent and constrained refinement
Δρ_max_ = 0.29 e Å^−3^
Δρ_min_ = −0.21 e Å^−3^

### 

Data collection: *CrysAlisPro* (Oxford Diffraction, 2009 ▶); cell refinement: *CrysAlisPro*; data reduction: *CrysAlisPro*; program(s) used to solve structure: *SIR2002* (Burla *et al.*, 2003 ▶); program(s) used to refine structure: *SHELXL97* (Sheldrick, 2008 ▶); molecular graphics: *ORTEP-3* (Farrugia, 1997 ▶); software used to prepare material for publication: *PLATON* (Spek, 2009 ▶).

## Supplementary Material

Crystal structure: contains datablocks global, I. DOI: 10.1107/S1600536809043001/wn2355sup1.cif

Structure factors: contains datablocks I. DOI: 10.1107/S1600536809043001/wn2355Isup2.hkl

Additional supplementary materials:  crystallographic information; 3D view; checkCIF report

## Figures and Tables

**Table 1:** Hydrogen-bond geometry (Å, °)

*D*—H⋯*A*	*D*—H	H⋯*A*	*D*⋯*A*	*D*—H⋯*A*
O1*W*—H1*W*⋯O7^i^	0.86 (3)	2.52 (3)	3.1550 (14)	131.9 (19)
O1*W*—H1*W*⋯N9^i^	0.86 (3)	2.16 (3)	2.9655 (15)	157 (2)
O1*W*—H2*W*⋯N1^ii^	0.88 (3)	2.05 (3)	2.9222 (15)	176 (2)
N8—H8⋯O1*W*	0.914 (18)	1.944 (18)	2.8486 (15)	170.3 (17)
C2—H2⋯O7^iii^	0.95	2.33	3.2253 (17)	157
C4—H4⋯O1*W*	0.95	2.54	3.2392 (16)	130
C10—H10⋯O7^i^	0.95	2.57	3.1507 (17)	120
C22—H22⋯*Cg*1^iv^	0.95	2.94	3.7742 (16)	148
C5—H5⋯*Cg*2^v^	0.95	2.54	3.4342 (15)	157

Symmetry codes: (i) 
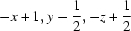
; (ii) 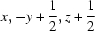; (iii) 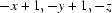; (iv) 
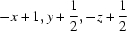
; (v) 
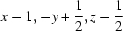
. *Cg*1 and *Cg*2 are the centroids of the N1–C6 and C21–C26 rings, respectively.

**Fig. 1.**
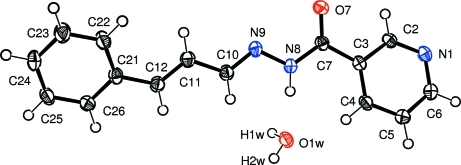
The molecular structure of the asymmetric unit, showing the atom-numbering scheme and displacement ellipsoids drawn at the 50% probability level. H atoms are shown as small spheres of arbitrary radius.

**Fig. 2.**
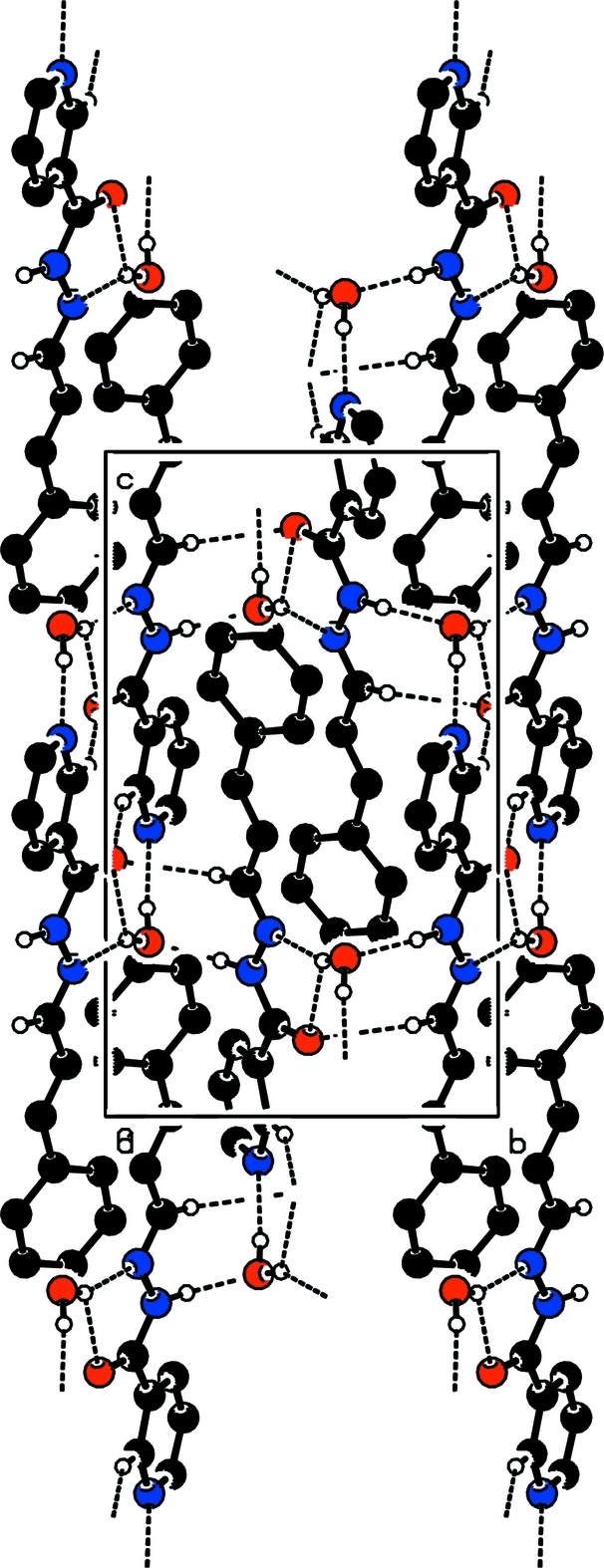
The packing of the title compound, viewed down the a axis. Dashed lines indicate hydrogen bonds. H atoms not involved in hydrogen bonding have been omitted.

## supplementary crystallographic information

### Comment

As part of our research, we have synthesized the title compound and report its crystal structure here. Archana *et al.* (2009) have reported a related crystal structure, *N*'-(2-methyl-3-phenylallylidene) nicotinohydrazide monohydrate.

The molecular structure of the asymmetric unit is shown in Fig. 1. The dihedral angle between the pyridine ring and the phenyl ring is 35.45 (7)°. Intermolecular O—H···O, O—H···N, N—H···O and C—H···O hydrogen bonds are found in the crystal structure. Furthermore, a C22—H22···π interaction involving the pyridine (N1—C6) ring and a C5—H5···π interaction involving the phenyl (C21—C26) ring are also found.

### Experimental

Sodium hydroxide (0.4 g, 0.01 mol) in a stoppered conical flask was kept in an ice-cold environment. Ethanol (40 ml) was added to dissolve it and the mixture was stirred continuously using a magnetic stirrer. An equimolar quantity of nicotinic hydrazide (1.371 g, 0.01 mol) and cinnamaldehyde (1.32 g, 0.01 mol) was added to this mixture. The stirring was continued for 5 h in ice-cold conditions. The mixture was kept overnight in a refrigerator. The mixture was then allowed to stand for four days under normal conditions. A yellow solid was obtained. This was filtered, washed and recrystallized from ethanol. Yield 2.3 g, 46.80%.

### Refinement

H8 attached to N8, and H1W and H2W attached to O1W were located in a difference Fourier map and refined freely. The remaining H atoms were positioned geometrically and allowed to ride on their parent atoms, with C—H = 0.95 Å. *U*_iso_(H) = 1.2*U*_eq_(C).

### Crystal data

**Table:**

C_15_H_13_N_3_O·H_2_O	*F*(000) = 568
*M**_r_* = 269.30	*D*_x_ = 1.288 Mg m^−^^3^
Monoclinic, *P*2_1_/*c*	Melting point: 463 K
Hall symbol: -P 2ybc	Cu *K*α radiation, λ = 1.54184 Å
*a* = 9.8456 (3) Å	Cell parameters from 3706 reflections
*b* = 9.1288 (3) Å	θ = 4.5–74.0°
*c* = 15.5389 (5) Å	µ = 0.72 mm^−^^1^
β = 95.938 (3)°	*T* = 110 K
*V* = 1389.12 (8) Å^3^	Plate, colourless
*Z* = 4	0.48 × 0.45 × 0.24 mm

### Data collection

**Table:**

Oxford Diffraction Xcalibur, Ruby, Gemini diffractometer	2742 independent reflections
Radiation source: Enhance (Cu) X-ray Source	2346 reflections with *I* > 2σ(*I*)
graphite	*R*_int_ = 0.022
Detector resolution: 10.5081 pixels mm^-1^	θ_max_ = 74.6°, θ_min_ = 4.5°
ω scans	*h* = −12→12
Absorption correction: multi-scan (*CrysAlis PRO*; Oxford Diffraction, 2009)	*k* = −10→9
*T*_min_ = 0.704, *T*_max_ = 1.000	*l* = −13→18
6007 measured reflections	

### Refinement

**Table:**

Refinement on *F*^2^	Primary atom site location: structure-invariant direct methods
Least-squares matrix: full	Secondary atom site location: difference Fourier map
*R*[*F*^2^ > 2σ(*F*^2^)] = 0.043	Hydrogen site location: inferred from neighbouring sites
*wR*(*F*^2^) = 0.125	H atoms treated by a mixture of independent and constrained refinement
*S* = 1.05	*w* = 1/[σ^2^(*F*_o_^2^) + (0.0821*P*)^2^ + 0.2609*P*] where *P* = (*F*_o_^2^ + 2*F*_c_^2^)/3
2742 reflections	(Δ/σ)_max_ = 0.001
193 parameters	Δρ_max_ = 0.29 e Å^−^^3^
0 restraints	Δρ_min_ = −0.20 e Å^−^^3^

### Special details

**Table:**

Geometry. Bond distances, angles *etc*. have been calculated using the rounded fractional coordinates. All su's are estimated from the variances of the (full) variance-covariance matrix. The cell e.s.d.'s are taken into account in the estimation of distances, angles and torsion angles
Refinement. Refinement of *F*^2^ against ALL reflections. The weighted *R*-factor *wR* and goodness of fit *S* are based on *F*^2^, conventional *R*-factors *R* are based on *F*, with *F* set to zero for negative *F*^2^. The threshold expression of *F*^2^ > 2σ(*F*^2^) is used only for calculating *R*-factors(gt) *etc*. and is not relevant to the choice of reflections for refinement. *R*-factors based on *F*^2^ are statistically about twice as large as those based on *F*, and *R*- factors based on ALL data will be even larger.

### Fractional atomic coordinates and isotropic or equivalent isotropic displacement parameters (Å^2^)

**Table:**

	*x*	*y*	*z*	*U*_iso_*/*U*_eq_	
O7	0.47479 (10)	0.51591 (11)	0.11459 (6)	0.0278 (3)	
N1	0.16249 (12)	0.38852 (13)	−0.06770 (7)	0.0251 (3)	
N8	0.40129 (11)	0.37087 (13)	0.21906 (7)	0.0229 (3)	
N9	0.50761 (11)	0.41751 (13)	0.27801 (7)	0.0239 (3)	
C2	0.26809 (14)	0.41542 (15)	−0.00816 (8)	0.0234 (4)	
C3	0.26600 (13)	0.38761 (14)	0.08017 (8)	0.0213 (3)	
C4	0.14754 (14)	0.32800 (15)	0.10770 (8)	0.0245 (4)	
C5	0.03714 (14)	0.30162 (16)	0.04673 (9)	0.0266 (4)	
C6	0.04847 (14)	0.33422 (15)	−0.03908 (9)	0.0249 (4)	
C7	0.39013 (13)	0.43067 (15)	0.13894 (8)	0.0214 (3)	
C10	0.51203 (13)	0.35650 (15)	0.35280 (9)	0.0244 (4)	
C11	0.61459 (14)	0.39886 (15)	0.42168 (9)	0.0253 (4)	
C12	0.61879 (13)	0.33520 (16)	0.49986 (8)	0.0252 (4)	
C21	0.71252 (14)	0.36867 (15)	0.57704 (8)	0.0241 (4)	
C22	0.79857 (16)	0.49073 (16)	0.58318 (9)	0.0308 (4)	
C23	0.89016 (17)	0.51244 (18)	0.65604 (10)	0.0366 (5)	
C24	0.89795 (15)	0.41399 (17)	0.72450 (9)	0.0319 (4)	
C25	0.80997 (15)	0.29477 (17)	0.72029 (9)	0.0306 (4)	
C26	0.71789 (14)	0.27272 (17)	0.64743 (9)	0.0273 (4)	
O1W	0.26480 (10)	0.11005 (11)	0.26235 (6)	0.0266 (3)	
H2	0.34889	0.45566	−0.02707	0.0281*	
H4	0.14252	0.30588	0.16702	0.0294*	
H5	−0.04511	0.26171	0.06373	0.0320*	
H6	−0.02844	0.31734	−0.08004	0.0299*	
H8	0.3493 (18)	0.294 (2)	0.2342 (11)	0.035 (5)*	
H10	0.44733	0.28292	0.36291	0.0293*	
H11	0.67948	0.47225	0.41157	0.0304*	
H12	0.55372	0.26002	0.50588	0.0302*	
H22	0.79430	0.55943	0.53706	0.0369*	
H23	0.94833	0.59572	0.65915	0.0440*	
H24	0.96260	0.42804	0.77357	0.0383*	
H25	0.81272	0.22804	0.76735	0.0366*	
H26	0.65768	0.19119	0.64543	0.0328*	
H1W	0.335 (3)	0.054 (3)	0.2663 (14)	0.057 (6)*	
H2W	0.232 (3)	0.106 (3)	0.3128 (16)	0.070 (7)*	

### Atomic displacement parameters (Å^2^)

**Table:**

	*U*^11^	*U*^22^	*U*^33^	*U*^12^	*U*^13^	*U*^23^
O7	0.0289 (5)	0.0327 (5)	0.0219 (5)	−0.0068 (4)	0.0028 (4)	0.0027 (4)
N1	0.0305 (6)	0.0268 (6)	0.0179 (5)	0.0013 (5)	0.0022 (4)	−0.0001 (4)
N8	0.0222 (5)	0.0268 (6)	0.0192 (6)	−0.0023 (4)	−0.0006 (4)	0.0013 (4)
N9	0.0247 (5)	0.0273 (6)	0.0191 (5)	−0.0010 (4)	−0.0004 (4)	−0.0012 (4)
C2	0.0267 (6)	0.0245 (7)	0.0194 (6)	0.0002 (5)	0.0046 (5)	0.0002 (5)
C3	0.0246 (6)	0.0204 (6)	0.0189 (6)	0.0021 (5)	0.0023 (5)	0.0008 (5)
C4	0.0263 (7)	0.0281 (7)	0.0194 (6)	0.0018 (5)	0.0032 (5)	0.0042 (5)
C5	0.0245 (6)	0.0295 (7)	0.0259 (7)	−0.0008 (5)	0.0029 (5)	0.0033 (5)
C6	0.0263 (6)	0.0242 (7)	0.0235 (7)	0.0020 (5)	−0.0007 (5)	−0.0009 (5)
C7	0.0231 (6)	0.0233 (6)	0.0182 (6)	0.0019 (5)	0.0036 (5)	−0.0004 (5)
C10	0.0249 (6)	0.0262 (7)	0.0219 (7)	0.0004 (5)	0.0014 (5)	0.0000 (5)
C11	0.0267 (7)	0.0260 (7)	0.0230 (7)	−0.0003 (5)	0.0013 (5)	−0.0019 (5)
C12	0.0241 (6)	0.0274 (7)	0.0236 (7)	−0.0002 (5)	0.0008 (5)	−0.0009 (5)
C21	0.0251 (6)	0.0268 (7)	0.0203 (6)	0.0040 (5)	0.0023 (5)	−0.0016 (5)
C22	0.0424 (8)	0.0250 (7)	0.0233 (7)	−0.0025 (6)	−0.0042 (6)	0.0028 (5)
C23	0.0461 (9)	0.0304 (8)	0.0310 (8)	−0.0089 (7)	−0.0074 (7)	−0.0004 (6)
C24	0.0352 (8)	0.0385 (8)	0.0202 (7)	0.0001 (6)	−0.0053 (6)	−0.0034 (6)
C25	0.0338 (7)	0.0383 (8)	0.0194 (6)	0.0016 (6)	0.0021 (5)	0.0044 (5)
C26	0.0263 (6)	0.0332 (7)	0.0229 (7)	−0.0011 (5)	0.0044 (5)	0.0011 (5)
O1W	0.0283 (5)	0.0294 (5)	0.0220 (5)	0.0024 (4)	0.0023 (4)	0.0048 (4)

### Geometric parameters (Å, °)

**Table:**

O7—C7	1.2283 (16)	C21—C26	1.3980 (19)
O1W—H1W	0.86 (3)	C22—C23	1.387 (2)
O1W—H2W	0.88 (3)	C23—C24	1.389 (2)
N1—C2	1.3410 (17)	C24—C25	1.388 (2)
N1—C6	1.3443 (18)	C25—C26	1.390 (2)
N8—N9	1.3848 (15)	C2—H2	0.9500
N8—C7	1.3534 (17)	C4—H4	0.9500
N9—C10	1.2854 (18)	C5—H5	0.9500
N8—H8	0.914 (18)	C6—H6	0.9500
C2—C3	1.3981 (18)	C10—H10	0.9500
C3—C4	1.3938 (19)	C11—H11	0.9500
C3—C7	1.5004 (18)	C12—H12	0.9500
C4—C5	1.3871 (19)	C22—H22	0.9500
C5—C6	1.382 (2)	C23—H23	0.9500
C10—C11	1.4461 (19)	C24—H24	0.9500
C11—C12	1.3435 (19)	C25—H25	0.9500
C12—C21	1.4678 (18)	C26—H26	0.9500
C21—C22	1.397 (2)		
			
O1W···N1^i^	2.9222 (15)	C22···H11	2.8000
O1W···O7^ii^	3.1550 (14)	C23···H5^ix^	2.9900
O1W···N9^ii^	2.9655 (15)	C24···H5^ix^	3.0700
O1W···C4	3.2392 (16)	C25···H5^ix^	2.9900
O1W···N8	2.8486 (15)	C26···H5^ix^	2.8000
O7···C10^iii^	3.1507 (17)	H1W···N9^ii^	2.16 (3)
O7···N9	2.6814 (14)	H1W···H8	2.26 (3)
O7···C2^iv^	3.2253 (17)	H1W···O7^ii^	2.52 (3)
O7···O1W^iii^	3.1550 (14)	H1W···C10^ii^	3.09 (3)
O1W···H10	2.7500	H2···O7	2.4700
O1W···H8	1.944 (18)	H2···O7^iv^	2.3300
O1W···H4	2.5400	H2W···N1^i^	2.05 (3)
O7···H2	2.4700	H2W···C2^i^	2.78 (2)
O7···H10^iii^	2.5700	H2W···H8	2.46 (3)
O7···H12^iii^	2.9100	H4···O1W	2.5400
O7···H2^iv^	2.3300	H4···N8	2.6600
O7···H26^v^	2.6200	H4···H8	2.1900
O7···H1W^iii^	2.52 (3)	H5···C21^x^	2.6900
N1···O1W^v^	2.9222 (15)	H5···C22^x^	2.8000
N8···O1W	2.8486 (15)	H5···C23^x^	2.9900
N9···O1W^iii^	2.9655 (15)	H5···C24^x^	3.0700
N9···O7	2.6814 (14)	H5···C25^x^	2.9900
N1···H2W^v^	2.05 (3)	H5···C26^x^	2.8000
N8···H26^v^	2.9300	H6···H24^viii^	2.4800
N8···H4	2.6600	H6···H23^ii^	2.5400
N9···H1W^iii^	2.16 (3)	H8···H1W	2.26 (3)
N9···H26^v^	2.8400	H8···O1W	1.944 (18)
C2···O7^iv^	3.2253 (17)	H8···C4	2.664 (17)
C4···O1W	3.2392 (16)	H8···H10	2.1300
C4···C24^vi^	3.576 (2)	H8···H2W	2.46 (3)
C5···C6^vii^	3.429 (2)	H8···H4	2.1900
C6···C22^ii^	3.575 (2)	H10···O7^ii^	2.5700
C6···C6^vii^	3.4332 (19)	H10···O1W	2.7500
C6···C23^ii^	3.539 (2)	H10···H8	2.1300
C6···C5^vii^	3.429 (2)	H10···H12	2.3700
C10···C22^vi^	3.594 (2)	H11···H22	2.2900
C10···O7^ii^	3.1507 (17)	H11···C22	2.8000
C10···C21^vi^	3.5874 (19)	H12···H10	2.3700
C21···C10^vi^	3.5874 (19)	H12···H26	2.3800
C22···C10^vi^	3.594 (2)	H12···O7^ii^	2.9100
C22···C6^iii^	3.575 (2)	H22···C11	2.8000
C23···C6^iii^	3.539 (2)	H22···H11	2.2900
C24···C4^vi^	3.576 (2)	H22···C6^iii^	2.9500
C2···H2W^v^	2.78 (2)	H23···C6^iii^	2.8700
C4···H8	2.664 (17)	H23···H6^iii^	2.5400
C6···H23^ii^	2.8700	H24···C6^xi^	3.0700
C6···H22^ii^	2.9500	H24···H6^xi^	2.4800
C6···H24^viii^	3.0700	H26···H12	2.3800
C7···H26^v^	2.8500	H26···O7^i^	2.6200
C10···H1W^iii^	3.09 (3)	H26···N8^i^	2.9300
C11···H22	2.8000	H26···N9^i^	2.8400
C21···H5^ix^	2.6900	H26···C7^i^	2.8500
C22···H5^ix^	2.8000		
			
H1W—O1W—H2W	106 (2)	C21—C26—C25	121.01 (14)
C2—N1—C6	116.98 (11)	N1—C2—H2	118.00
N9—N8—C7	117.93 (11)	C3—C2—H2	118.00
N8—N9—C10	114.67 (11)	C5—C4—H4	121.00
C7—N8—H8	123.5 (11)	C3—C4—H4	121.00
N9—N8—H8	118.1 (11)	C4—C5—H5	120.00
N1—C2—C3	123.71 (12)	C6—C5—H5	120.00
C2—C3—C7	117.14 (11)	C5—C6—H6	118.00
C4—C3—C7	124.77 (11)	N1—C6—H6	118.00
C2—C3—C4	118.03 (12)	N9—C10—H10	120.00
C3—C4—C5	118.66 (12)	C11—C10—H10	120.00
C4—C5—C6	119.07 (13)	C12—C11—H11	120.00
N1—C6—C5	123.52 (13)	C10—C11—H11	120.00
N8—C7—C3	115.96 (11)	C11—C12—H12	116.00
O7—C7—C3	120.96 (11)	C21—C12—H12	116.00
O7—C7—N8	123.08 (12)	C23—C22—H22	120.00
N9—C10—C11	120.60 (12)	C21—C22—H22	120.00
C10—C11—C12	120.49 (13)	C22—C23—H23	120.00
C11—C12—C21	127.36 (13)	C24—C23—H23	120.00
C12—C21—C22	123.19 (12)	C25—C24—H24	120.00
C12—C21—C26	118.59 (12)	C23—C24—H24	120.00
C22—C21—C26	118.21 (12)	C24—C25—H25	120.00
C21—C22—C23	120.55 (13)	C26—C25—H25	120.00
C22—C23—C24	120.84 (15)	C21—C26—H26	119.00
C23—C24—C25	119.11 (14)	C25—C26—H26	119.00
C24—C25—C26	120.21 (13)		
			
C6—N1—C2—C3	1.2 (2)	C3—C4—C5—C6	0.4 (2)
C2—N1—C6—C5	−2.0 (2)	C4—C5—C6—N1	1.2 (2)
C7—N8—N9—C10	179.89 (12)	N9—C10—C11—C12	179.83 (13)
N9—N8—C7—O7	5.25 (19)	C10—C11—C12—C21	−177.76 (13)
N9—N8—C7—C3	−174.34 (11)	C11—C12—C21—C22	10.7 (2)
N8—N9—C10—C11	−177.62 (12)	C11—C12—C21—C26	−168.23 (14)
N1—C2—C3—C4	0.3 (2)	C12—C21—C22—C23	−176.56 (14)
N1—C2—C3—C7	−177.10 (12)	C26—C21—C22—C23	2.4 (2)
C2—C3—C4—C5	−1.10 (19)	C12—C21—C26—C25	176.51 (13)
C7—C3—C4—C5	176.05 (13)	C22—C21—C26—C25	−2.5 (2)
C2—C3—C7—O7	16.54 (19)	C21—C22—C23—C24	−0.3 (2)
C2—C3—C7—N8	−163.87 (12)	C22—C23—C24—C25	−1.7 (2)
C4—C3—C7—O7	−160.64 (13)	C23—C24—C25—C26	1.6 (2)
C4—C3—C7—N8	18.96 (19)	C24—C25—C26—C21	0.5 (2)

Symmetry codes: (i) *x*, −*y*+1/2, *z*+1/2; (ii) −*x*+1, *y*−1/2, −*z*+1/2; (iii) −*x*+1, *y*+1/2, −*z*+1/2; (iv) −*x*+1, −*y*+1, −*z*; (v) *x*, −*y*+1/2, *z*−1/2; (vi) −*x*+1, −*y*+1, −*z*+1; (vii) −*x*, −*y*+1, −*z*; (viii) *x*−1, *y*, *z*−1; (ix) *x*+1, −*y*+1/2, *z*+1/2; (x) *x*−1, −*y*+1/2, *z*−1/2; (xi) *x*+1, *y*, *z*+1.

### Hydrogen-bond geometry (Å, °)

**Table:**

*D*—H···*A*	*D*—H	H···*A*	*D*···*A*	*D*—H···*A*
O1W—H1W···O7^ii^	0.86 (3)	2.52 (3)	3.1550 (14)	131.9 (19)
O1W—H1W···N9^ii^	0.86 (3)	2.16 (3)	2.9655 (15)	157 (2)
O1W—H2W···N1^i^	0.88 (3)	2.05 (3)	2.9222 (15)	176 (2)
N8—H8···O1W	0.914 (18)	1.944 (18)	2.8486 (15)	170.3 (17)
C2—H2···O7^iv^	0.95	2.33	3.2253 (17)	157
C4—H4···O1W	0.95	2.54	3.2392 (16)	130
C10—H10···O7^ii^	0.95	2.57	3.1507 (17)	120
C22—H22···Cg1^iii^	0.95	2.94	3.7742 (16)	148
C5—H5···Cg2^x^	0.95	2.54	3.4342 (15)	157

Symmetry codes: (ii) −*x*+1, *y*−1/2, −*z*+1/2; (i) *x*, −*y*+1/2, *z*+1/2; (iv) −*x*+1, −*y*+1, −*z*; (iii) −*x*+1, *y*+1/2, −*z*+1/2; (x) *x*−1, −*y*+1/2, *z*−1/2.

## References

[bb1] Archana, R., Manimekalai, A., Saradhadevi, N., Thiruvalluvar, A. & Butcher, R. J. (2009). *Acta Cryst.* E**65**, o1659. 10.1107/S1600536809023368 PMC2969494 21582921

[bb2] Burla, M. C., Camalli, M., Carrozzini, B., Cascarano, G. L., Giacovazzo, C., Polidori, G. & Spagna, R. (2003). *J. Appl. Cryst.* **36**, 1103.

[bb3] Farrugia, L. J. (1997). *J. Appl. Cryst.* **30**, 565.

[bb4] Oxford Diffraction (2009). *CrysAlisPro* Oxford Diffraction Ltd, Abingdon, England.

[bb5] Sheldrick, G. M. (2008). *Acta Cryst.* A**64**, 112–122. 10.1107/S0108767307043930 18156677

[bb6] Spek, A. L. (2009). *Acta Cryst.* D**65**, 148–155. 10.1107/S090744490804362X PMC2631630 19171970

